# The identification of high-performing antibodies for Profilin-1 for use in Western blot, immunoprecipitation and immunofluorescence

**DOI:** 10.12688/f1000research.132249.1

**Published:** 2023-03-29

**Authors:** Riham Ayoubi, Ian McDowell, Maryam Fotouhi, Kathleen Southern, Peter S. McPherson, Carl Laflamme, NeuroSGC/YCharOS/EDDU collaborative group, ABIF Consortium

**Affiliations:** 1 Department of Neurology and Neurosurgery, Structural Genomics Consortium, The Montreal Neurological Institute, McGill University, Montreal, Quebec, H3A 2B4, Canada

**Keywords:** Uniprot ID P07737, PFN1, Profilin-1, antibody characterization, antibody validation, Western blot, immunoprecipitation, immunofluorescence

## Abstract

Profilin-1, a member of the Profilin family, is a ubiquitously expressed protein that controls actin polymerization in a concentration-dependent manner. As mutations in the Profilin-1 gene have potential implications in neurodegenerative disease progression, well-characterized anti-Profilin-1 antibodies would be beneficial to the scientific community. In this study, we characterized sixteen Profilin-1 commercial antibodies for Western blot, immunoprecipitation, and immunofluorescence applications, using a standardized experimental protocol based on comparing read-outs in knockout cell lines and isogenic parental controls. We identified many high-performing antibodies and encourage readers to use this report as a guide to select the most appropriate antibody for their specific needs.

## Introduction

Profilin-1, encoded by the *PFN1* gene, is ubiquitously expressed and the most abundant of the Profilin genes. ^1^ It plays an essential role in the polymerization of actin by binding and sequestering actin monomers, indicating its cytoskeletal function. ^2^^,^^3^ At high concentrations, Profilin-1 act as an inhibitor of actin polymerization whereas at low concentrations, it acts as a catalyst. ^3^^,^^4^

Profilin-1 is of recent interest thanks to the identification of *PFN1* mutations in 25 human familial amyotrophic lateral sclerosis (fALS) patients. ^5^^–^^8^ *PFN1* is among a group of ALS-related genes that directly affect cytoskeletal dynamics, namely *TUBA4, ALS2, KIF5A* and *SPAST*, hypothesizing that cytoskeletal dysfunction contributes to motor neuron degeneration. ^9^ *PFN1* mutant mice that carry the G118V mutation have motor defects consistent with ALS pathology, suggesting that Profilin-1 molecular studies would provide insight into pathogenic mechanisms of motor neuron disease. ^10^ Mechanistic studies would be greatly facilitated with the availability of high-quality antibodies.

Here, we compared the performance of a range of high-quality commercially available antibodies for Profilin-1 and characterized several antibodies for Western blot, immunoprecipitation and immunofluorescence experiments, enabling biochemical and cellular assessment of Profilin-1 properties and function.

## Results and discussion

Our standard protocol involved comparing readouts from wild-type (WT) and knockout (KO) cells. ^11^^–^^13^ The first step was to identify a cell line(s) that expressed sufficient levels of Profilin-1 to generate a measurable signal. To this end, we examined the DepMap transcriptomics database to identify all cell lines that express the target at levels greater than 2.5 log _2_ (transcripts per million “TPM” + 1), which we found to be a suitable cut-off (Cancer Dependency Map Portal, RRID:SCR_017655). Of all cell lines analyzed, commercially available HAP1 cells expressed the *PFN1* RNA transcript above the adequate cut-off level. Parental and *PFN1* KO HAP1 cells were obtained from Horizon Discovery ( Table 1).

**Table 1.** Summary of the cell lines used.

Institution	Catalog number	RRID (Cellosaurus)	Cell line	Genotype
Horizon Discovery	C631	CVCL_Y019	HAP1	WT
Horizon Discovery	HZGHC005831c016	CVCL_C4J6	HAP1	*PFN1* KO

**Figure 1.**
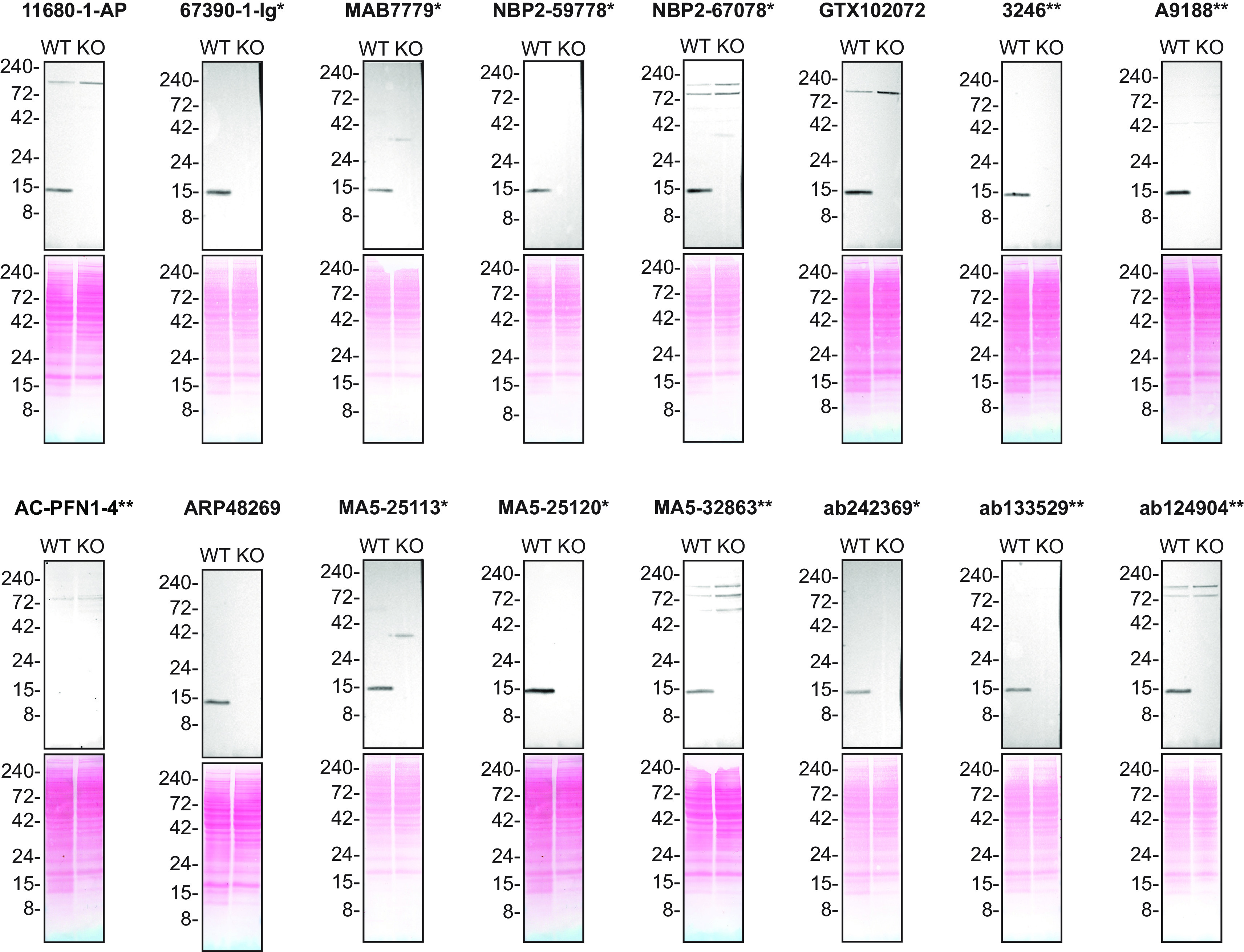
Profilin-1 antibody screening by Western blot. Lysates of HAP1 (WT and *PFN1* KO) were prepared and 50 μg of protein were processed for Western blot with the indicated Profilin-1 antibodies. The Ponceau stained transfers of each blot are presented to show equal loading of WT and KO lysates and protein transfer efficiency from the polyacrylamide gels to the nitrocellulose membrane. Antibody dilutions were chosen according to the recommendations of the antibody supplier. An exception was given for antibody A9188** which was titrated to 1/30000, as the signal was too weak when following the supplier’s recommendation. When the concentration was not indicated by the supplier, which was the case for AC-PFN1-4**, we tested the antibody at 1/1000. Antibody dilution used: 11680-1-AP at 1/6000, 67390-1-lg* at 1/30000, MAB7779* at 1/5000, NBP2-59778* at 1/1000, NBP2-67078* at 1/2000, GTX102072 at 1/3000, 3246** at 1/1000, A9188 at 1/30000, AC-PFN1-4** at 1/1000, ARP48269 at 1/2000, MA5-25113* at 1/2000, MA5-25120* at 1/2000, MA5-32683** at 1/2000, ab242369* at 1/2000, ab133529* at 1/10000, ab124904** at 1/30000. Predicted band size: 15 kDa. *Monoclonal antibody, **Recombinant antibody.

For Western blot experiments, we resolved proteins from WT and *PFN1* KO cell extracts and probed them side-by-side with all antibodies in parallel ^12^^,^^13^ ( Figure 1).

**Figure 2.**
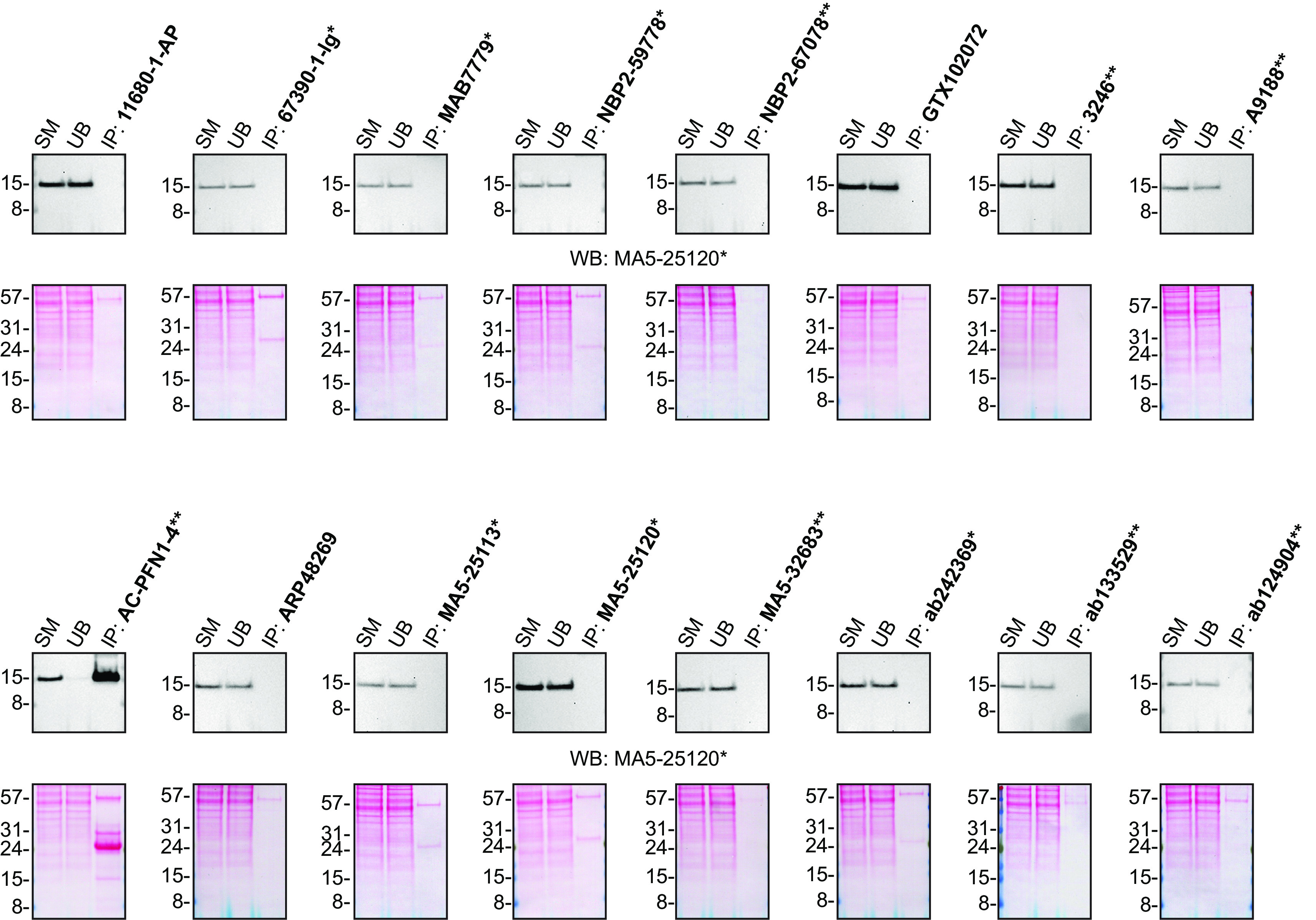
Profilin-1 antibody screening by immunoprecipitation. HAP1 lysates were prepared, and IP was performed using 2.0 μg of the indicated Profilin-1 antibodies pre-coupled to Dynabeads protein G or protein A or Flag-M2 magnetic beads. Samples were washed and processed for Western blot with the indicated Profilin-1 antibody. For Western blot, MA5-25120* was used at 1/5000. The Ponceau stained transfers of each blot are shown for similar reasons as in Figure 1. SM=2% starting material; UB=2% unbound fraction; IP=Immunoprecipitate. *Monoclonal antibody, **Recombinant antibody.

For immunoprecipitation experiments, we used the antibodies to immunopurify Profilin-1 from HAP1 WT cell extracts. The performance of each antibody was evaluated by detecting the Profilin-1 protein in extracts, in the immunodepleted extracts and in the immunoprecipitates ^12^^,^^13^ ( Figure 2).

**Figure 3.**
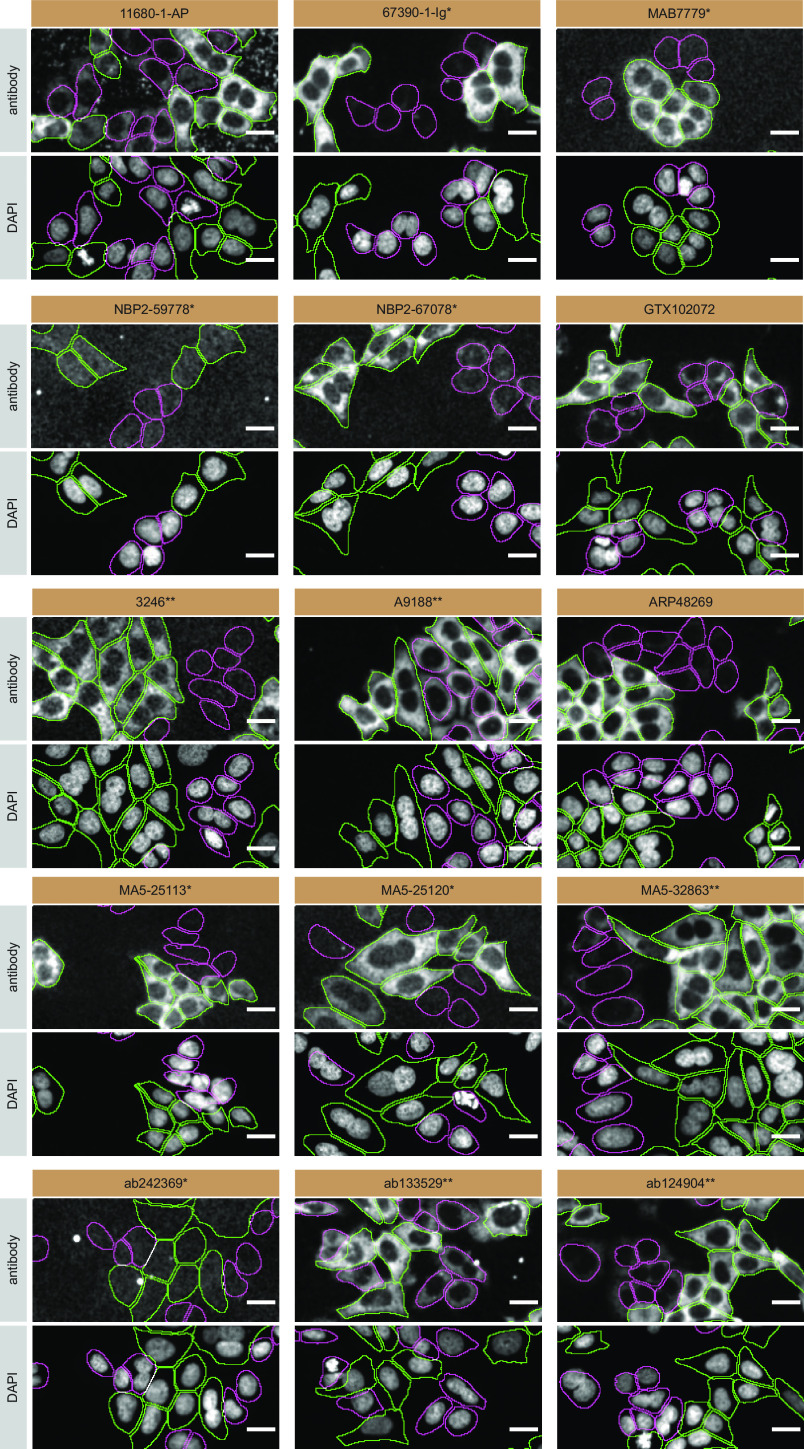
Profilin-1 antibody screening by immunofluorescence. HAP1 WT and *PFN1* KO cells were labelled with a green or a far-red fluorescent dye, respectively. WT and KO cells were mixed and plated to a 1:1 ratio in a 96-well glass plate. Cells were stained with the indicated Profilin-1 antibodies and with the corresponding Alexa-fluor 555 coupled secondary antibody including DAPI. Acquisition of the blue (nucleus-DAPI), green (identification of WT cells), red (antibody staining) and far-red (identification of KO cells) channels was performed. Representative images of the merged blue and red (grayscale) channels are shown. WT and KO cells are outlined with green and magenta dashed line, respectively. Primary antibodies were tested at 1.0 μg/mL. Antibody dilutions were chosen according to the recommendations of the antibody supplier. Exceptions were given for antibodies 67390-1-Ig*, NBP2-67078*, MA5-25113*, MA5-25120* and MA5-32683** which were titrated as the signals were too weak when following the supplier’s recommendations. When the concentrations were not indicated by the supplier, which was the case for antibodies 11680-1-AP, MAB7779*, NBP2-59778*, 3246**, A9188**, ARP48269, ab242369* and ab133529*, we tested antibodies at 1/400, 1/1000, 1/500, 1/100, 1/3000,1/500, 1/500 and 1/60, respectively. At these concentrations, the signal from each antibody was in the range of detection of the microscope used. Antibody dilution used: 11680-1-AP at 1/400, 67390-1-lg* at 1/2000, MAB7779* at 1/1000, NBP2-59778* at 1/500, NBP2-67078* at 1/1000, GTX102072 at 1/500, 3246** at 1/100, A9188** at 1/3000, ARP48269 at 1/500, MA5-25113* at 1/1000, MA5-25120* at 1/600, MA5-32683** at 1/1000, ab242369* at 1/500, ab133529* at 1/60, ab124904** at 1/150. Bars=10 μm. *Monoclonal antibody, **Recombinant antibody.

For immunofluorescence, as described previously, antibodies were screened using a mosaic strategy. ^12^^–^^14^ In brief, we plated WT and KO cells together in the same well and imaged both cell types in the same field of view to reduce staining, imaging and image analysis bias ( Figure 3).

In conclusion, we have screened Profilin-1 commercial antibodies by Western blot, immunoprecipitation and immunofluorescence and identified several high-quality antibodies using our standardized experimental conditions. The underlying data can be found on Zenodo. ^15^^,^^16^

## Methods

### Antibodies

All Profilin-1 antibodies are listed in Table 2, together with their corresponding Research Resource Identifiers (RRID), to ensure the antibodies are cited properly. ^17^ Peroxidase-conjugated goat anti-rabbit and anti-mouse antibodies are from Thermo Fisher Scientific (cat. number 65-6120 and 62-6520). Peroxidase-conjugated monoclonal anti-Flag M2 is from MilliporeSigma (cat. number A8592). Alexa-555-conjugated goat anti-mouse and anti-rabbit secondary antibodies are from Thermo Fisher Scientific (cat. number A21424 and A21429). The anti-FLAG (M2 clone) conjugated with Cy3 is from MilliporeSigma (cat. number A9594).

**Table 2.** Summary of the Profilin-1 antibodies tested.

Company	Catalog number	Lot number	RRID (Antibody Registry)	Clonality	Clone ID	Host	Concentration (μg/μL)	Vendors recommended applications
Proteintech	11680-1-AP	44905	AB_2163182	polyclonal	-	rabbit	0.37	Wb, IF
Proteintech	67390-1-lg *	10014183	AB_2924436 ^1^	monoclonal	3A12E8	mouse	2.3	Wb, IF
Bio-Techne	MAB7779 *	CHHC0112101	AB_2885149	monoclonal	816536	mouse	1.0	Wb
Bio-Techne	NBP2-59778 *	MAB-03023	AB_2885159	monoclonal	CL3524	mouse	0.5	Wb
Bio-Techne	NBP2-67078 *	HK0713	AB_2827372	monoclonal	JA30-13	rabbit	1.0	Wb, IF
GeneTex	GTX102072	40660	AB_1241179	polyclonal	-	rabbit	0.56	Wb, IF
Cell Signaling Technology	3246 **	1	AB_2163185	recombinant-mono	C56B8	rabbit	n/a	Wb
ABclonal	A9188 **	106890101	AB_2863682	recombinant-mono	-	rabbit	2.79	Wb
Structural Genomics Consortium	AC-PFN1-4 ** ^a^	YSPFN1A-c002	N/A	recombinant-mono	AC-PFN1-4	human	1.13	IP
Aviva Systems Biology	ARP48269	QC20112-42607	AB_1294625	polyclonal	-	rabbit	0.5	Wb
Thermo Fisher Scientific	MA5-25113 *	VL3152362	AB_2725111	monoclonal	OTI1D5	mouse	1.0	Wb, IF
Thermo Fisher Scientific	MA5-25120 *	VL3152363	AB_2725112	monoclonal	OTI2D2	mouse	0.58	Wb, IF
Thermo Fisher Scientific	MA5-32683 **	VL3152615	AB_2809960	recombinant-mono	JA30-13	rabbit	1.0	Wb, IF
Abcam	ab242369 *	GR3380758-1	AB_2885122	monoclonal	CL3524	mouse	0.5	Wb
Abcam	ab133529 *	GR98050-4	AB_2885087	recombinant-mono	EPR6303	rabbit	0.057	Wb
Abcam	ab124904 **	GR3233753-5	AB_10975882	recombinant-mono	EPR6304	rabbit	0.143	Wb, IF

Wb=Western blot; IF= immunofluorescence; IP=immunoprecipitation.

* Monoclonal antibody.

** Recombinant antibody.

^1^ Refer to RRID recently added to the Antibody Registry (on January 2023), they will be available on the Registry website in coming weeks.

^a^ AC-PFN1-4** sequence: EVQLLESGGGLVQPGGSLRLSCAASGFTFYSSYMYWVRQAPGKGLEWVSSISGYGGYTYYADSVKGRFTISRDNSKNTLYLQMNSLRAEDTAVYYCARWDGYMDYWGQGTLVTVSSGGGGSGGGGSGGGGSDIQMTQSPSSLSASVGDRVTITCRASQSISSYLNWYQQKPGKAPKLLIYAASSLQSGVPSRFSGSGSGTDFTLTISSLQPEDFATYYCQQYWDYGLPTFGQGTKLEIK https://www.eubopen.org/antibodies.

### Cell culture

Both HAP1 WT and *PFN1* KO cell lines used are listed in Table 1, together with their corresponding Research Resource Identifiers (RRID), to ensure the cell lines are cited properly. ^18^ Cells were cultured in DMEM high glucose (GE Healthcare, cat. number SH30081.01) containing 10% fetal bovine serum (Wisent, cat. number 080450), 2 mM L-glutamate (Wisent, cat. number 609065), 100 IU penicillin and 100 μg/mL streptomycin (Wisent, cat. number 450201).

### Antibody screening by Western blot

Western blots were performed as described in our standard operating procedure. ^19^ HAP1 WT and *PFN1* KO were collected in RIPA buffer (25 mM Tris-HCl pH 7.6, 150 mM NaCl, 1% NP-40, 1% sodium deoxycholate, 0.1% SDS) (Thermo Fisher Scientific, cat number 0089901) supplemented with 1× protease inhibitor cocktail mix (MilliporeSigma, cat. number 78429). Lysates were sonicated briefly and incubated for 30 min on ice. Lysates were spun at ~110,000× g for 15 min at 4°C and equal protein aliquots of the supernatants were analyzed by SDS-PAGE and Western blot. BLUelf prestained protein ladder (GeneDireX, cat. number PM008-0500) was used.

Western blots were performed with precast midi 10% Bis-Tris polyacrylamide gels (Thermo Fisher Scientific, cat. number WG1201BOX) ran with MES SDS buffer (Thermo Fisher Scientific, cat. number NP000202), loaded in LDS sample buffer (Thermo Fisher Scientific, cat. number NP0008) with 1× sample reducing agent (Thermo Fisher Scientific, cat. number NP0009) and transferred on nitrocellulose membranes. Proteins on the blots were visualized with Ponceau staining which is scanned to show them together with individual Western blots. Blots were blocked with 5% milk for 1 hr, and antibodies were incubated overnight at 4°C with 5% bovine serum albumin (BSA) (Wisent, cat. number 800-095) in TBS with 0.1% Tween 20 (TBST) (Cell Signalling, cat. number 9997). Following three washes with TBST, the peroxidase conjugated secondary antibody was incubated at a dilution of ~0.2 μg/mL in TBST with 5% milk for 1 hr at room temperature followed by three washes with TBST. Membranes were incubated with Pierce ECL from Thermo Fisher Scientific (cat. number 32106) prior to detection with the iBright™ CL1500 Imaging System from Thermo Fisher Scientific (cat. number A44240).

### Antibody screening by immunoprecipitation

Immunoprecipitation was performed as described in our standard operating procedure. ^20^ Antibody-bead conjugates were prepared by adding 2 μg or 20 μL of antibody at an unknown concentration to 500 μL of Pierce IP Lysis Buffer from Thermo Fisher Scientific (cat. number 87788) in a 1.5 mL microcentrifuge tube, together with 30 μL of Dynabeads protein A- (for rabbit antibodies) or protein G- (for mouse antibodies) from Thermo Fisher Scientific (cat. number 10002D and 10004D, respectively) or anti-Flag M2 magnetic beads from MilliporeSigma (cat. number M8823). Tubes were rocked for ~1 hr at 4°C followed by two washes to remove unbound antibodies.

HAP1 WT were collected in Pierce IP buffer (25 mM Tris-HCl pH 7.4, 150 mM NaCl, 1 mM EDTA, 1% NP-40 and 5% glycerol) from Thermo Fisher Scientific (cat. number 87788), supplemented with protease inhibitor from MilliporeSigma (cat. number P8340). Lysates were rocked 30 min at 4°C and spun at 110,000× g for 15 min at 4°C. 0.5 mL aliquots at 2.0 mg/mL of lysate were incubated with an antibody-bead conjugate for ~1 hr at 4°C. Following centrifugation, the unbound fractions were collected, and beads were subsequently washed three times with 1.0 mL of IP lysis buffer and processed for SDS-PAGE and Western blot on a precast midi 10% Bis-Tris polyacrylamide gels as described above.

### Antibody screening by immunofluorescence

Immunofluorescence was performed as described in our standard operating procedure. ^12^^–^^14^ HAP1 WT and *TMEM106B* KO were labelled with CellTracker ^TM^ green (Thermo Fisher Scientific, cat. number C2925) or CellTracker ^TM^ deep red (Thermo Fisher Scientific, cat. number C34565) fluorescence dye, respectively. The nuclei were labelled with DAPI (Thermo Fisher Scientific, cat. number D3571) fluorescent stain. WT and KO cells were plated in 96 well glass plates (Perkin Elmer, cat. number 6055300) as a mosaic and incubated for 24 hrs in a cell culture incubator at 37 ^o^C, 5% CO _2_. Cells were fixed in 4% paraformaldehyde (PFA) (Beantown chemical, cat. number 140770-10ml) in phosphate buffered saline (PBS) (Wisent, cat. number 311-010-CL) for 15 min at room temperature and then washed three times with PBS. Cells were permeabilized in PBS with 0.1% Triton X-100 (Thermo Fisher Scientific, cat. number BP151-500) for 10 min at room temperature and blocked with PBS with 5% BSA, 5% goat serum (Gibco, cat. number 16210-064) and 0.01% Triton X-100 for 30 min at room temperature. Cells were incubated with IF buffer (PBS, 5% BSA, 0.01% Triton X-100) containing the primary Profilin-1 antibodies overnight at 4°C. Cells were then washed three times for 10 min with IF buffer and incubated with corresponding Alexa Fluor 555-conjugated secondary antibodies in IF buffer at a dilution of 1.0 μg/mL for 1 hr at room temperature with DAPI. Cells were washed three times for 10 min with IF buffer and once with PBS.

Images were acquired on an ImageXpress micro confocal high-content microscopy system (Molecular Devices), using a 20× NA 0.95 water immersion objective and scientific CMOS cameras (16-bit, 1.97mm field of view), equipped with 395, 475, 555 and 635 nm solid state LED lights (Lumencor Aura III light engine) and bandpass emission filters (432/36 nm, 520/35 nm, 600/37 nm, 692/40 nm) to excite DAPI, CellTracker ^TM^ Green, Alexa fluor 555 and CellTracker ^TM^ Red, respectively. Images had pixel sizes of 0.68 × 0.68 microns. Exposure time was set with maximal (relevant) pixel intensity, ~80% of dynamic range and verified on multiple wells before acquisition. Since the IF staining varied depending on the primary antibody used, the exposure time was set using the most intensely stained well as reference. Frequently, the focal plane varied slightly within a single field of view. To remedy this issue, a stack of three images per channel was acquired at a z-interval of 4 microns per field and best focus projections were generated during the acquisition ( MetaXpress v6.7.1, Molecular Devices). Segmentation was carried out on the projections of CellTracker ^TM^ channels using CellPose v1.0 on green (WT) and far-red (KO) channels, using as parameters the ‘cyto’ model to detect whole cells, and using an estimated diameter tested for each cell type, between 15 and 20 microns. ^21^ Masks were used to generate cell outlines for intensity quantification. Background was subtracted using a minimum projection of all images collected in the Alexa fluor 555 channel. Images shown for the Alexa fluor 555 channel were modified by applying a Gaussian blur filter with a radius value of one pixel. Figures were first assembled with Adobe Photoshop (version 24.2.1) to adjust contrast then finally assembled with Adobe Illustrator (version 27.3.1).

## Data Availability

Zenodo: Antibody Characterization Report for Profilin-1, https://doi.org/10.5281/zenodo.7249258. ^15^ Zenodo: Dataset for the Profilin-1 antibody screening study, https://doi.org/10.5281/zenodo.7671118. ^16^ Data are available under the terms of the Creative Commons Attribution 4.0 International license (CC-BY 4.0)
