# Supplementary material for: The identification of high-performing antibodies for Profilin-1 for use in Western blot, immunoprecipitation and immunofluorescence
Source: F1000Res. 2023 Mar 29;12:348. [Version 1] doi: 10.12688/f1000research.132249.1 (PMC10415725; doi:10.12688/f1000research.132249.1)
Supplement: Peer review history

# Peer review: Reviewer response for version 1

**DOI:** 10.5256/f1000research.145159.r178173

**Published:** 2023-08-10

**Authors:** Xiaoyan Du

**Affiliations:** 1 Capital Medical University, Beijing, China

This article described a work to identify high-performing antibodies for Profilin-1 using 16 Profilin-1 commercial antibodies by Western blot, immunoprecipitation and immunofluorescence analysis. It is a very significant job for those who investigate PFN1 function in medical and biological field. I concerned following issues which need explanation by authors:

1. All WB experiments are lack of reference such as GAPDH etc. when tested PFN1. Therefore, the readers could not know if the difference among the bands detected by each kind of antibodies caused by various commercial antibodies or the technique.
2. As to test antibodies, at least two kind of cell lines are needed. The introduction should give readers more information about PFN1 function in more diseases, not just focus on neurodegenerative disease.

Are sufficient details of methods and materials provided to allow replication by others?

Yes

Is the rationale for creating the dataset(s) clearly described?

Yes

Are the datasets clearly presented in a useable and accessible format?

Yes

Are the protocols appropriate and is the work technically sound?

Yes

Reviewer Expertise:

NA

I confirm that I have read this submission and believe that I have an appropriate level of expertise to confirm that it is of an acceptable scientific standard, however I have significant reservations, as outlined above.

# Peer review: Reviewer response for version 1

**DOI:** 10.5256/f1000research.145159.r183160

**Published:** 2023-07-18

**Authors:** Jieya Shao

**Affiliations:** 1 Washington University, St. Louis, USA

This is an excellent study containing high-quality data and detailed experimental procedures. The paper is clearly written and figures are nicely presented with high levels of technical detail allowing replication by others. The findings are highly informative for investigators interested in profilin-1, particularly with regard to its antibodies for immunofluorescence staining and immunoprecipitation purposes.

 The only suggestion would be to clarify the purpose of using the anti-FLAG M2 magnetic beads for immunoprecipitation experiments. Which antibodies were characterized using these beads and why?

Are sufficient details of methods and materials provided to allow replication by others?

Yes

Is the rationale for creating the dataset(s) clearly described?

Yes

Are the datasets clearly presented in a useable and accessible format?

Yes

Are the protocols appropriate and is the work technically sound?

Yes

Reviewer Expertise:

cell biology, cancer biology, genome integrity maintenance

I confirm that I have read this submission and believe that I have an appropriate level of expertise to confirm that it is of an acceptable scientific standard.

# Peer review: Reviewer response for version 1

**DOI:** 10.5256/f1000research.145159.r168349

**Published:** 2023-04-14

**Authors:** Partha Roy

**Affiliations:** 1 Department of Bioengineering, University of Pittsburgh, Pittsburgh, PA, USA

- It is a well-executed study, and a good resource for researchers working on Pfn1.
- I would have liked to see some conclusions with regard to the recommendation of antibodies for specific purposes based on the findings of the study.
- Table 2: concentration is listed as microgram/microliter - should it not be microgram/ml?

Are sufficient details of methods and materials provided to allow replication by others?

Yes

Is the rationale for creating the dataset(s) clearly described?

Yes

Are the datasets clearly presented in a useable and accessible format?

Yes

Are the protocols appropriate and is the work technically sound?

Yes

Reviewer Expertise:

cell biology, cancer biology

I confirm that I have read this submission and believe that I have an appropriate level of expertise to confirm that it is of an acceptable scientific standard, however I have significant reservations, as outlined above.

# Response

**Published:** 2023-04-14

**Authors:** Kathleen Southern

**Affiliations:** McGill University, Canada

Thank you to Partha Roy (reviewer #1) for your review and further contribution to this study for Profilin-1.

 Dr. Roy believes our study would be improved if we drew conclusions from the data and provided antibody recommendations for the different applications. After presenting the YCharOS initiative, which aims to characterize antibodies for human proteins to dissolve the reproducibility crisis, on several occasions, we’ve found that for the most part, scientists interested in our reports have the expertise to interpret the antibody characterization data. Moreover, we do not score/recommend antibodies because we tested the antibodies under one set of conditions, and the scoring/recommendation is only valid under this precise experimental setup and in the cell line used. That being said, YCharOS reports serve as an invaluable guide pointing scientists to appropriate antibodies for their experimental needs.

 Dr. Roy also asks whether the concentration units listed in Table 2 should be in mg/mL rather than μg/μl. To that we would like to elucidate the fact that both units of concentration are equivalent. Using mg/mL is equivalent to using μg/μl.

 Thank you again to Partha Roy for your suggestions!
